# Entrepreneurial Passion to Entrepreneurial Behavior: Role of Entrepreneurial Alertness, Entrepreneurial Self-Efficacy and Proactive Personality

**DOI:** 10.3389/fpsyg.2020.01611

**Published:** 2020-08-20

**Authors:** Cai Li, Majid Murad, Fakhar Shahzad, Muhammad Aamir Shafique Khan, Sheikh Farhan Ashraf, Courage Simon Kofi Dogbe

**Affiliations:** School of Management, Jiangsu University, Zhenjiang, China

**Keywords:** entrepreneurial passion, entrepreneurial alertness, entrepreneurial self-efficacy, proactive personality, entrepreneurial intention, entrepreneurial behavior

## Abstract

This study investigated the role of entrepreneurial passion in recognition of opportunity, developing entrepreneurial self-efficacy, and entrepreneurial intention, in the shaping of entrepreneurial actions in the presence of proactive personality. This study applied partial least squares structural equation modeling to test the hypotheses on a sample of 346 university students from Jiangsu province, China. The output of the study showed that entrepreneurial passion positively and significantly influenced entrepreneurial alertness, entrepreneurial self-efficacy to entrepreneurial intention, and entrepreneurial behavior. The findings also showed that a proactive personality positively and significantly moderated the relationship between entrepreneurial intention and entrepreneurial behavior.

## Introduction

Entrepreneurship has a recognized impact on a country’s economic growth, job creation, and innovation (Laguía et al., 2019; Cardella et al., 2020). Several governments and private sectors depend on entrepreneurial start-ups for employment creation (Sánchez-García et al., 2018; Neneh, 2019a). With an increasing number of individuals studying and completing university education in China, appropriate job searching has become a serious concern for graduate students. Therefore, students have been motivated by universities to start new businesses to release employment pressure for university students. According to Hu and Ye (2017), in developed countries the entrepreneurship success rate is 20%, as compared to Chinese university graduates where only 2% of students start their businesses with a 10% success rate. Prior studies have investigated the drivers of entrepreneurship by identifying why individuals develop the entrepreneurial intention to become an entrepreneur (Kautonen et al., 2015; Fuller et al., 2018; Bueckmann-Diegoli and Gutiérrez, 2020). These studies primarily used entrepreneurial models to explain the formation of entrepreneurial intention with very limited attention paid to the role of entrepreneurial behavior activity. It is therefore imperative to move beyond models that end at enlightening intentions, to move toward how these entrepreneurial intentions convert into entrepreneurial behavioral actions (Shirokova et al., 2016; Shinnar et al., 2018), as the decision mechanisms that enable an individual’s entrepreneurial behavior remain an open issue in the field of behavioral study.

Entrepreneurial intention refers to the intent to formulate a new business as well as select an alternate career to common employment (Ward et al., 2019a; Yi, 2020). As suggested by several authors, intentions are the best predictor for measuring entrepreneurial behavior (Fitzsimmons and Douglas, 2011; Ajzen and Sheikh, 2013). Prior researchers found that individuals with a high level of entrepreneurial intentions have a positive and significant influence on entrepreneurial behavior (Kautonen et al., 2015; Neneh, 2019a). Over the past three decades, numerous studies investigated the influence of entrepreneurial passion on predicting entrepreneurial intention and entrepreneurial orientation (Cardon and Kirk, 2015; Campos, 2017). Less attention has been paid to the influence of entrepreneurial passion in the formation of entrepreneurial behavior. The effect of entrepreneurial passion with the impact of entrepreneurial alertness, entrepreneurial self-efficacy, and proactive personality on entrepreneurial behavior have been given limited attention in the literature. Therefore, to address this gap, we have tried to measure the relationship between intention and behavior in order to illustrate their link and contribute more to the existing literature of entrepreneurship (Fuller et al., 2018; Hu et al., 2018; Karimi, 2020). Looking into existing literature, we found that intentions do not always lead to entrepreneurial action formation in the conceptual model of the intention–behavior gap in the entrepreneurship field (Van Gelderen et al., 2008). Some scholars found that in the conceptualization of intention and behavior model, entrepreneurial intentions explain no more than 30% of the variance in entrepreneurial action (Shirokova et al., 2016; Neneh, 2019a).

Consequently, we still lack the understanding of what kind of influences translates intentions into actions in the entrepreneurial background, particularly in the relationship between entrepreneurial passion, entrepreneurial self-efficacy, entrepreneurial alertness, and proactive personality. This research is still under-explored and in its development stage (Bogatyreva et al., 2019; Yi, 2020). Thus, it is essential to connect all these indicators to provide new theoretical and practical insights. To tackle this issue in a timely manner, we have used a university student sample from two Chinese universities to investigate the extent to which predictors are linked with entrepreneurial intentions which translate into entrepreneurial behavior.

The structure of the paper is as follows. After the introduction, we presented a discussion on theoretical and hypotheses development. This was followed by the materials, methods, measurement, and structural model. We also discussed the findings of previous researchers and then concluded with theoretical and practical implications and limitations for future research directions.

## Literature Review and Hypothesis Development

### Entrepreneurial Passion and Entrepreneurial Intention

Entrepreneurial passion is associated with positive feelings and attitudes for activities that are crucial to the self-identity of the individual (Huyghe et al., 2016). Passion is considered as the heart of entrepreneurship and it can become a vital component of entrepreneurial behavior action as well as the business creation process and its outcomes (Cardon et al., 2013; Santos and Cardon, 2019). According to Vallerand et al. (2003), passion is associated with the strong feeling to perform any task that individuals like to achieve with full energy. Research has widely shown that entrepreneurial passion plays a significant role in entrepreneurial intention (Biraglia and Kadile, 2017; Schenkel et al., 2019; Karimi, 2020). Moreover, some scholars identified that entrepreneurial passion developed positive feelings among individuals and improved motivational factors when the environment was uncertain and resources were narrow (De Clercq et al., 2013; Moses et al., 2016; Türk et al., 2020).

Cardon et al. (2017) suggested that entrepreneurial passion motivates people to spot innovative opportunities and develop a new business intention. Similarly, Hubner et al. (2019) stated that entrepreneurial passion is the crucial factor for achieving motivation and success and is important in predicting entrepreneurial intention. Karimi (2020) examined the direct role of entrepreneurial passion and the mediating effect of the theory of planned behavior using 310 Iranian public university students’ data from different courses. The results illustrate that entrepreneurial passion and theory of planned behavior helped students in forming entrepreneurial intentions. Additionally, Campos (2017) investigated an empirical study on entrepreneurial passion and the mediating role of entrepreneurial alertness using 112 technology-based entrepreneurs’ samples. The findings indicate that entrepreneurial passion and entrepreneurial alertness positively and significantly influenced entrepreneurial orientation.

Existing literature focused on the entrepreneur’s traits and how these traits affect entrepreneurial passion, business success, and owners’ decisions (Cardon et al., 2009; Sánchez, 2011; Fellnhofer, 2017) Sánchez, 2011). Scholars explained the three types of entrepreneurial passion which are related to several characteristics of the entrepreneurial process. Firstly, passion regarding entrepreneurship and involvement in identifying, inventing, and exploring new opportunities. Secondly, passion for founding reflects the entrepreneur’s passion regarding activities involved in making a business venture and the related marketing and opportunity exploitation activities. Thirdly, passion regarding care, forecasting, progress, and expanding the venture after it is established (Cardon and Kirk, 2015; Campos, 2017). These entrepreneurial passions connected to identity influence and to achieving the entrepreneurial intention. Therefore, individuals with a high level of entrepreneurial passion are more likely to form a business and translate their passion into action. Hence, the study will propose the following hypothesis:

**H1.** Entrepreneurial passion will have a positive influence on entrepreneurial intention.

### Entrepreneurial Passion and Entrepreneurial Alertness

Entrepreneurial alertness is associated with the identification of opportunities and exploitation (Kirzner, 1985; Obschonka et al., 2017; Neneh, 2019a). Entrepreneurial alertness is the inclination of an individual to create a positive image of future outcomes (Uy et al., 2015). Moreover, Kirzner (1997) articulated that alertness refers to the entrepreneurs’ cognitive abilities and skills that direct the process of opportunity identification in the market. Gozukara and Colakoglu (2016) explained that entrepreneurial alertness is a state of mind that is open to exploring opportunities at all times, even in uncertain environments with limited resources.

Tang et al. (2012) suggested that the ability to make the relation between dots by linking them on different occasions is a dynamic feature of recognizing opportunities. Previous studies on entrepreneurial alertness were empirically tested by authors (Kaish and Gilad, 1991; Busenitz, 1996) and it was found that there is a problem with the conceptual measurement model, although a similar situation persists in empirical estimations related to entrepreneurial alertness (Demmert and Klein, 2003; Kitzmann and Schiereck, 2005). Researchers developed the three dimensions of entrepreneurial alertness: alert scanning and search refers to information accumulation which an individual has during the identification of the opportunity discovery process in the market. This comprises both prior and fresh knowledge which is related to the individual’s characteristics and information to the specific fields which enable him/her to discover the innovative opportunities; Aalert association and connections refer to information transformation, which relates to collecting information on different environments and using that knowledge to form new substitutes; and alert evaluation and judgment refers to information selection, which involves making assessments and judgments about new information and deciding whether there is a potentially profitable opportunity (Tang et al., 2012; Amato et al., 2017). This relates to preparing people to select appropriate patterns from multiple patterns concurrently. Consequently, this alertness allows the individual to choose the relevant information in a better situation, which improves the possibility to explore the perfect opportunity. However, previous researchers suggested that entrepreneurial alertness is linked with prior knowledge, information handling abilities, social relationships, opportunity recognition, and entrepreneurial generosity (Foo, 2011; Renko et al., 2012; Ho et al., 2018).

Furthermore, Hayton and Cholakova (2012) identified that entrepreneurial passion influences idea awareness and supports the development of entrepreneurial intention. Entrepreneurial passion stimulates the processeses involved in opportunity evaluation and information memory to make effective and efficient decisions (Foo, 2011; Welpe et al., 2012). Likewise, Campos (2017) found that entrepreneurial passion enables the formation of unfamiliar associations, recognition of opportunity, and encourages entrepreneurs to involve themselves with novel ideas to articulate creative paths of action. Thus, individuals with a high level of entrepreneurial passion for becoming entrepreneurs are more likely to be alert for the recognition of opportunity. Therefore, the study will assume the following hypothesis:

**H2.** Entrepreneurial passion will have a positive influence on entrepreneurial alertness.

### Entrepreneurial Passion and Entrepreneurial Self-Efficacy

The ability to start a new business based on an individual’s own skill and ability is defined as self-efficacy (Boyd and Vozikis, 1994; Stroe et al., 2018). Self-efficacy is the core component of social cognitive theory (SCT), which stimulates the willingness of individuals to accomplish their responsibilities and achieve their expectations. Self-efficacy is thought to be a very perspective-specific quality, leading to a higher outcome-forecasting rate when customized to a common activity context (Bandura, 1982; Drnovšek et al., 2014). A higher level of entrepreneurial passion and the ability to establish innovative business solutions seem to be the foundations of having entrepreneurial intentions (Cardon et al., 2013; Cardon and Kirk, 2015). Prior studies have shown that passionate entrepreneurs are driven toward committed identification of new opportunities, which is essential for entrepreneurial intention (Cardon et al., 2005; Jarvis, 2016). Nonetheless, starting a business will pose multiple challenges related to establishing a company. Individuals might see challenges as hurdles, but they may be more and more eager to overcome them by developing innovative and alternate ideas. Consequently, business people must depend on the assumption that they can perform and reach their full potential with their expertise (Dalborg and Wincent, 2015; Biraglia and Kadile, 2017).

Moreover, entrepreneurial self-efficacy is characterized by the self-belief that skills can be adopted to achieve goal-oriented tasks (Barbosa et al., 2007; Nowiński et al., 2019). As it shapes a complex series of mutually reinforcing expectations of one’s ability to complete a project or achieve an objective, entrepreneurial self-efficacy tends to be a very significant criterion for business intentions (Zhao et al., 2005). Baron (2008) believed that self-efficacy modifies the cognitive process, and explained that perception depends on different thinking and outlining probabilistic patterns guiding the decision-making process to pursue a career in entrepreneurship (Mannino and Faraci, 2017). In many cases, new ideas cause people to rethink their ability to engage in innovation in the context of starting a new business full of entrepreneurial passion (Bagheri and Yazdanpanah, 2017). Therefore, a high degree of passion also contributes to a new venture start-up. Individuals tend to see themselves as capable of becoming an entrepreneur. Thus, this study will predict the following hypothesis:

**H3.** Entrepreneurial passion will have a positive influence on entrepreneurial self-efficacy.

### Entrepreneurial Alertness and Entrepreneurial Intention

Prior studies found that entrepreneurial alertness has a positive relationship with entrepreneurial intention (Tang et al., 2012; Hu et al., 2018; Neneh, 2019a). Entrepreneurial alertness has received important attention in the field of entrepreneurship, and it identifies the appropriate career and exploitation of entrepreneurial opportunities (Tang, 2008; Short et al., 2010). The process starts with the ability to explore the opportunity, and the endless development by the individual to turn that opportunity into a reality (Campos, 2017). According to McMullen and Shepherd (2006), entrepreneurial alertness must generate entrepreneurial action. However, entrepreneurial intention refers to a self-acknowledged belief to start a new business in the future (Kautonen et al., 2013). Entrepreneurial intention includes the original concept idea and the plan to formulate and start new ventures as per entrepreneurial choice (Obschonka et al., 2017; Mannino et al., 2019). Researchers have shown that entrepreneurial intention plays a dynamic part in the building of an individual’s entrepreneurial behavior to begin his/her own business (Van Gelderen et al., 2008; Shinnar et al., 2018). As per the theory of planned behavior, the stronger an individual’s intention to involve in a specific behavior, the more likely it is that the real behavior will be reflected in the organization (Ajzen, 1991). Lu and Wang (2018) confirmed that entrepreneurial alertness has directly influenced entrepreneurial intention because entrepreneurial alertness develops the recognition of opportunity and judgment identification of individuals with business intent.

Furthermore, some scholars identified that alertness is a crucial skill for entrepreneurs to predict and identify opportunities (Ardichvili and Cardozo, 2000; Mannino and Schiera, 2017; Shamsudeen et al., 2017). Hu et al. (2018) examined the role of entrepreneurial alertness with the help of SCT and recognized that entrepreneurial alertness is an important predictor for examining entrepreneurial intention. Thus, several studies confirmed that entrepreneurial alertness positively and significantly mediates between entrepreneurial intention and entrepreneurial orientation (Li et al., 2015; Campos, 2017; Neneh, 2019a). Therefore, it is observed that individuals with a greater level of alertness could identify appropriate opportunities and pursue a new career in entrepreneurship. So, the study will forecast the following hypothesis:

**H4.** Entrepreneurial alertness will have a positive influence on entrepreneurial intention.

### Entrepreneurial Self-Efficacy and Entrepreneurial Intention

Previous researchers acknowledged that entrepreneurial self-efficacy is positively related to entrepreneurial intention (Naktiyok et al., 2010; Park and Choi, 2016; Tsai et al., 2016). Literature suggested that entrepreneurial self-efficacy is positively linked with the formation of entrepreneurial intention (Mauer et al., 2017). Entrepreneurial self-efficacy is also influenced by environmental factors, vicarious experience, and social modeling, which stand as both barriers and facilitators; thus, entrepreneurial self-efficacy signifies the emotional mechanism that is crucial for individuals to understand that they are skillful enough to perform different tasks and behaviors in a complex environment (Ciuchta and Finch, 2019). Moreover, the behavior dimension of SCT is associated with the outcomes of personal, environmental, and past experiences. Previous experiences and past behaviors could affect future entrepreneurial intentions and actions through an increase in entrepreneurial self-efficacy to become an entrepreneur in the future (McGee and Peterson, 2019).

However, individuals must have prior knowledge regarding start-up businesses in terms of what to do and how to run a successful business. Individuals tend to see themselves as capable of performing business-related behavior to cultivate business start-up intentions (Ward et al., 2019b). Thus, individuals with a high level of self-efficacy will be more prone to react positively in any emerging situation in contrast to those who have low self-efficacy, who believe they will always remain hesitant to any new environment (Zhao et al., 2005; Hsu et al., 2019). Therefore, this study will propose the following hypothesis:

**H5.** Entrepreneurial self-efficacy will have a positive influence on entrepreneurial intention.

### Entrepreneurial Self-Efficacy and Entrepreneurial Behavior

When examining the literature, it can be seen that a few empirical studies have been conducted on the relationship between entrepreneurial self-efficacy and entrepreneurial behavior. According to Ajzen (1991), theory of planned behavior defined intentions as an individual’s belief and perception of the performance of a specific behavior. Some scholars argued that entrepreneurial intention ultimately leads to entrepreneurial behavior (Shirokova et al., 2016; Yi, 2020). However, a recent study stated that entrepreneurial intention is the strongest predictor for entrepreneurial behavior (Neneh, 2019a). Entrepreneurs needs a strong individual commitment to translate entrepreneurial intentions into entrepreneurial behavior. Moreover, previous researchers illustrated that entrepreneurial action is a practice of entrepreneurial behavior as an understanding of the entrepreneurial intentions of entrepreneurship, and that entrepreneurial self-efficacy contributes to entrepreneurial intention and entrepreneurial behavior (Darmanto and Yuliari, 2018; Neneh, 2019b).

On the other hand, it has been established that entrepreneurs with extraordinary self-efficacy for a specific undertaking are more likely to follow through and continue in that activity than entrepreneurs who hold lesser self-efficacy (Darmanto and Yuliari, 2018). Zhao et al. (2005) exhibited an optimistic consequence relating to entrepreneurial self-efficacy upon the expansion of entrepreneurial behavior. Furthermore, specifically entrepreneurial self-efficacy has a constructive effect upon acknowledgment of prospect, enactment of a new project, and the entrepreneurial tasks of an individual. Therefore, individuals with a high level of self-efficacy are more prone to create a new business. Thus, we will predict this hypothesis:

**H6.** Entrepreneurial self-efficacy will have a positive impact on entrepreneurial behavior.

### Entrepreneurial Alertness and Entrepreneurial Behavior

Several studies have examined the relationship between entrepreneurial alertness and entrepreneurial intention (Campos, 2017; Obschonka et al., 2017; Hu et al., 2018). The relationship between entrepreneurial alertness and entrepreneurial behavior is less studied in the literature. A prior study suggested that entrepreneurial alertness to entrepreneurial behavior is an important part of the entrepreneurial process of starting a new business (Short et al., 2010). According to Sirén et al. (2019) alertness is associated with human motivation, which creates awareness among individuals to identify and recognize the opportunities to become entrepreneurs. Similarly, Hu and Ye (2017) explained that alertness is the process of pursuing the entrepreneurial opportunities which are siezed through procedures such as identification, exploitation, and evaluation of an opportunity, as well as attaining resources and planning a method to achieve this opportunity.

Moreover, a recent study by Neneh (2019a) examined the entrepreneurial intentions of students using entrepreneurial alertness as a predictor variable and found that entrepreneurial alertness had a positive and significant influence on entrepreneurial intention, however, the relationship between entrepreneurial alertness and entrepreneurial behavior is still under-explored. Furthermore, Obschonka et al. (2017) found that entrepreneurial alertness had a positive effect on entrepreneurial intention and career adaptability. Thus, individuals with a high level of alertness are more likely to identify and exploit opportunities to become entrepreneurs. Based on the above discussion, we have assumed that entrepreneurial alertness will develop an entrepreneurial behavior among individuals. Hence, we will propose this hypothesis:

**H7.** Entrepreneurial alertness will have a positive influence on entrepreneurial behavior.

### Entrepreneurial Intention and Entrepreneurial Behavior

There has been a vast amount of research on entrepreneurial behavior. Entrepreneurial intention is associated with an individual’s willingness to develop entrepreneurial behavior and commit to starting a new business (Ceresia and Mendola, 2019). Based on the theory of planned behavior and its extension of reasoned action theory, Ajzen (1991) defined entrepreneurial intention as individuals’ willingness to involve in entrepreneurial behavior or his/her commitment toward starting a new venture (Kautonen et al., 2015). This theory explained that there is a positive effect of entrepreneurial intention on entrepreneurial behavior, and further, it has been confirmed by researchers (Shinnar et al., 2018; Neneh, 2019a). Prior investigations acknowledged the importance of entrepreneurial intention models to understand the entrepreneurial phenomenon and proved it to be an effective indicator of measuring entrepreneurial behavior (Fayolle and Gailly, 2015; Shirokova et al., 2016).

Entrepreneurial intention provides strength and encourages the individual to engage in entrepreneurial behavior, and reflects the amount of effort that such a person is ready to commit to business development activities (Kristiansen and Indarti, 2004; Schlaegel and Koenig, 2014). However, many studies have examined the relationship between an individual’s entrepreneurial intentions and their entrepreneurial behavior (Kautonen et al., 2015; Lu and Wang, 2018; Neneh, 2019a), but the impact of entrepreneurial passion, entrepreneurial alertness, entrepreneurial self-efficacy, and a proactive personality on entrepreneurial intentions and behaviors has not yet been sufficiently examined. Existing literature provides a significant and positive contribution to the influence of entrepreneurial intention with the formulation of entrepreneurial behavior (Kautonen et al., 2013; Daim et al., 2016; Feola et al., 2019). Therefore, as discussed by the theory of planned behavior, individuals with a stronger level of entrepreneurial intent are more inclined to achieve actual behavior for new business development. Hence, the present study will assume the following hypothesis:

**H8.** Entrepreneurial intention will have a positive influence on entrepreneurial behavior.

### Proactive Personality and Entrepreneurial Behavior

Proactive personality is associated with active attempts made by the individual to effect changes in his or her environment (Delle and Amadu, 2016). Proactive personality plays a vital role in the formation of an entrepreneurial intention to entrepreneurial behavior, because the personality approach to entrepreneurship has progressively revealed that different personality traits are involved in shaping entrepreneurial intention and successive actions (Hu et al., 2018; Neneh, 2019b). Proactive personality individuals can identify opportunities and take the right actions at the right times (Mustafa et al., 2016; Marler et al., 2017). The role of proactive behavior in the organizational framework has observed it influences the attitude of the business owner toward searching for opportunities and competitive orientation (Viinikainen et al., 2017).

Studies show that many individuals who behave differently toward various environmental stimuli are more proactive and always carry out constructive development that could lead to a better environment (Chipeta and Surujlal, 2017; Fuller et al., 2018). Crant (1996) looked at the entrepreneurial intention of college students and stated that students who are more proactive are more likely to start their businesses. Hu et al. (2018) conducted a cross-sectional study to measure student’s entrepreneurial intentions with proactive personality and creativity in China. The results indicate that a proactive personality had a more significant and positive impact on entrepreneurial intention when compared to creativity. Travis and Freeman (2017) aimed to predict the entrepreneurial intention of 471 participants from a private college and public university in the Southeastern United States. The findings suggested that proactive personality behavior had a significant contribution to entrepreneurial self-efficacy and entrepreneurial intention.

Bateman and Crant (1993) constructed a scale for evaluating positive personality by examining the literature. Their study found that individuals with a high level of proactive personality are more inclined to achieve career success, work performance, and venture development. Furthermore, Prabhu et al. (2012) examined the cross-cultural review of businesses, from countries including Finland, China, the United States, and Russia, and the results explained that a proactive personality had a significant impact on entrepreneurial business intention. Thus, students with a proactive personality can easily identify opportunities in the market and persevere until a meaningful outcome ensues to start their businesses. Hence, the study will predict the following hypothesis:

**H9.** Proactive personality will have a positive influence on entrepreneurial behavior.

### The Moderating Role of Proactive Personality Between Entrepreneurial Intention and Entrepreneurial Behavior

Proactive personality related to the person who is able to perform in a manner that develops a change in their environment, regardless of any accessible constraints by situational conditions in the environment (Zampetakis, 2008; Laguía et al., 2019). Proactive personality refers to an individual’s tendency to take appropriate action and develop and acting on entrepreneurial intentions (Claes et al., 2005). Proactive personality individuals have a virtuous propensity to identify the opportunities in their struggles and to take the appropriate steps to overcome these opportunities until their planned target is accomplished (Kumar and Shukla, 2019). As suggested by prior studies, a proactive personality played a vital role in predicting entrepreneurial intention and entrepreneurial behavior (Van Gelderen et al., 2008; Neneh, 2019b). However, individuals with a high level of proactive personality are more persuaded to actively design and take the essential actions to achieve their targets.

Proactive personality individuals are seen as those that ultimately take action on the identification of good opportunities (Becherer and Maurer, 1999). As suggested by prior studies, numerous individuals have entrepreneurial intention but they are unsuccessful at converting them into actions due to environmental constraints (Fuller et al., 2018). Therefore, an individual with a proactive personality can remain persistent in achieving their targets without being swayed by uncertain situational factors that create the quality of a valuable moderator in the formation of entrepreneurial intentions to entrepreneurial actions. Thus, a proactive personality is an inspiring quality that enables individuals with intentions to take crucial actions to involve themselves in business development activities. Consequently, the study will propose the following hypothesis:

**H10.** Proactive personality will have a positive moderating effect between entrepreneurial intention and entrepreneurial behavior.

### Conceptual Model

**FIGURE 1:**
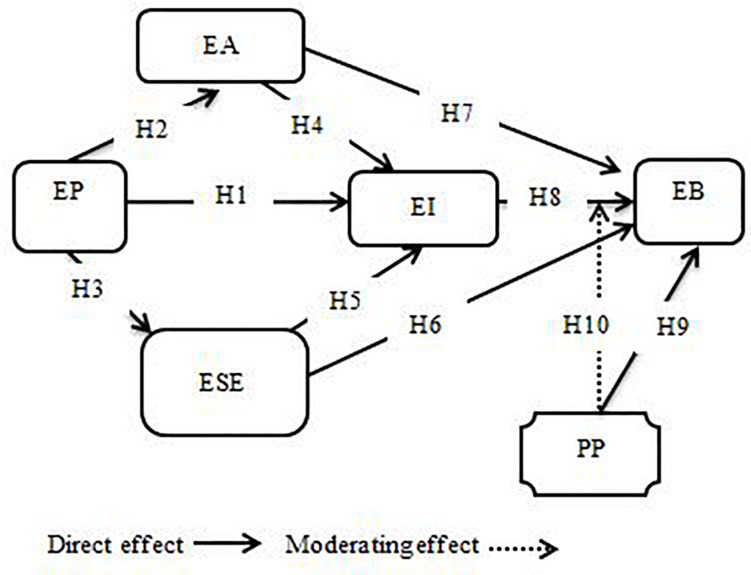
Conceptual Model.

In order to identify the influence of the direct relationship between entrepreneurial passion and entrepreneurial intention, as well as through entrepreneurial self-efficacy and entrepreneurial alertness, we have conceptualized the entrepreneurial behavior model with a moderating effect of proactive personality. Figure 1 shows the research framework for entrepreneurial behavior.

## Materials and Methods

This study was a survey and the data was collected using a close-ended questionnaire from students studying at two universities: Jiangsu University and Jiangsu University of Science and Technology, Zhenjiang city, Jiangsu province, China. The respondents were students, as there is an emerging trend of university graduates starting entrepreneurial ventures (Shirokova et al., 2016; Neneh, 2019b).

### Pilot Testing

Based on the existing validated scale, a preliminary questionnaire was formulated and a pilot study was conducted on 40 randomly selected volunteers enrolled in different business and management schools at universities in Jiangsu province, Zhenjiang city, China. The original questionnaire adapted was in the English language and first translated into the Chinese language using four different language experts. The Chinese version was translated back to the English language to ensure the validity and correctness of the main idea. The updated questionnaire was comprised of 47 items, and the respondents were asked to give their opinion on a five-point Likert scale, from 1 to 5 (1 referring to strongly disagree and 5 referring to strongly agree).

### Population and Sampling Technique

The targeted population included students of two universities in Jiangsu province, Zhenjiang city, China. The total population of the selected students was approximately 4,000 business students. According to Krejcie and Morgan (1970), if the target population is more than 4,000, a minimum sample size of 345 is enough. Moreover, a non-probability sampling technique (convenience sampling) was used to select respondents from universities in Jiangsu province, Zhenjiang city. According to Mason (2010), the formula for calculating the sample size *z*^2^ × *p*(1 - *p*)/*e*^2^, where *z* = 1.6384, *p* = 0.25, and *e*^2^ = 0.0016, the sample size for this study is approximately 350. Initially, 400 questionnaires were distributed, after the respondents were informed about the anonymity of their identities and full consent was taken. In total, 355 questionnaires were returned and the participation rate was 88.7%. Moreover, nine incomplete questionnaires were discarded, thus, the final response size compromised of 346 valid questionnaires. Among the valid questionnaires, 199 (57.5%) were filled by males and 147 (42.5%) by females. The age range was from 15 to 35 years, the mean age 2.02, and the standard deviation 0.820. There were 249 (72.0%) undergraduate students, 84 (24.3%) masters students, and 13 (3.8%) Ph.D. students. The study field of students was 181 (52.3%) from the school of management, 131 (37.9%) from the school of economics and finance, and 34 (9.8%) from public administration.

### Measures

#### Entrepreneurial Passion

The study adapted an entrepreneurial passion measurement scale from the study of Cardon et al., 2013 (see Appendix). The scale has five measurement constructs. A sample item is “I am motivated to figure out how to make existing products/services better.” A five-point scale was used ranging from 1 = strongly disagree to 5 = strongly agree.

#### Entrepreneurial Alertness

An entrepreneurial alertness measurement scale was adapted from the study by Tang et al., 2012. The scale contains thirteen constructs and is divided into three dimensions: scanning and search, association connections and evaluation, and judgment. A sample item for scanning and searching is “I always keep an eye out for new business ideas when looking for information.” For association with connections: “I am good at connecting dots.” For evaluation and judgment: “When facing multiple opportunities, I can select the good ones.” We have used a five-point scale ranging from 1 = strongly disagree to 5 = strongly agree.

#### Entrepreneurial Self-Efficacy

Entrepreneurial self-efficacy was measured using a four items scale developed by Zhao et al., 2005. A five-point scale was used, ranging from 1 = strongly disagree to 5 = strongly agree. A sample item for entrepreneurial self-efficacy is: “I am convinced that I can successfully discover new business opportunities.”

#### Entrepreneurial Intention

The entrepreneurial intention was measured using the five measurement constructs developed by Liñán and Chen, 2009 which is widely accepted and used by many researchers (Santos Cumplido et al., 2016; Neneh, 2019b). A five-point scale was used, ranging from 1 = strongly disagree to 5 = strongly agree. An entrepreneurial intention sample statement is, “I am ready to do anything to be an entrepreneur.”

#### Proactive Personality

Proactive personality had 17 measurement items that were developed by Bateman and Crant, 1993; this scale was reduced to a 10 item scale by previous researchers (Seibert et al., 1999). This scale was widely used and validated by past researchers (Gupta and Bhawe, 2007; Prabhu et al., 2012). Thus, this study also used this scale to measure proactive personality with 10 constructs. A sample item for proactive personality is, “Nothing is more exciting than seeing my ideas turn into reality.” All the constructs were measured using a five-point scale, ranging from 1 = strongly disagree to 5 = strongly agree.

#### Entrepreneurial Behavior

Entrepreneurial behavior was measured using a 10 items scale. The present study adapted the measurement constructs from the Global Entrepreneurship Monitor (GEM) and Panel Study of Entrepreneurship Dynamics (PSED), which developed a set of start-up activities regarding entrepreneurial behavior. The present study adopted the 10 measurement constructs scale from a prior study (Shirokova et al., 2016; Neneh, 2019b). A sample item is “I have purchased materials, equipment, or machinery for the business.” All the items were assessed using a five-point scale, ranging from 1 = strongly disagree to 5 = strongly agree.

#### Common Method Bias

Harman’s one-factor test was applied to the data (Harman, 1976). According to Harman’s methodology, if all the factors merged into one factor and it explains the >50% of the total variance, then there is a possibility of a common bias method (Podsakoff et al., 2003). The present study collected data from students because researchers already informed them regarding the purpose of the study. To check for common method bias, all the factors were introduced in one factor and the result of the first factor explained 20.79% of initial eigenvalues of the variance. Thus, there is no potential problem in common method variance.

## Results

### Data Analysis Method

The results were analyzed using partial least square-structural equation modeling (PLS-SEM) from Smart-PLS software version 3.0. The benefit of this software stems from its robustness in checking predictive applications, theory establishment, and explanation (Chin, 1998; Khan et al., 2019b). This software is used for the exploratory nature of study analysis and is also suitable for measuring mediating and moderating effects in the same path model. In recent times, this software has been considered a silver bullet in the field of management science and behavioral research (Hair et al., 2011; Shahzad et al., 2020).

### Measurement Model Analysis

Before performing the estimations, it is expected to assess the reliability and validity of the adapted measurement scales. Construct reliability and composite reliability were measured using α (Brown, 2002). As indicated in Table 1, the values of α and composite reliability were greater than the threshold value 0.70, suggested by Nunally and Bernstein, 1978; Khan et al., 2019a. Convergent validity was assessed using average variance extracted (AVE) values shown in Table 1; all the values were acceptable of the entire model following the commonly used threshold value of 0.5 (Henseler et al., 2016). The study also checked for the issue of multicollinearity, as suggested by Aiken et al., 1991; an outer Variance Inflation Factor (VIF) less than five considered excellent. Thus, all the measurement constructs VIF values were satisfactory and under the threshold.

**TABLE 1:** Factor Loadings.

Constructs	Loadings	Cronbach’s Alpha (CA)	Composite Reliability (CR)	Average Variance Extracted (AVE)	VIF
**Entrepreneurial Passion**		0.949	0.948	0.785	
EP 1	0.986				4.029
EP 2	0.790				4.567
EP 3	0.903				3.752
EP 4	0.861				3.982
EP 5	0.879				3.502
**Entrepreneurial Alertness**		0.958	0.957	0.636	
EA 1	0.764				4.001
EA 2	0.697				3.507
EA 3	0.908				2.899
EA 4	0.705				3.058
EA 5	0.792				3.557
EA 6	0.814				3.719
EA 7	0.841				3.343
EA 8	0.753				3.301
EA 9	0.828				3.407
EA 10	0.721				3.216
EA 11	0.912				3.359
EA 12	0.872				3.190
EA 13	0.715				3.106
**Entrepreneurial Self-Efficacy**		0.907	0.908	0.711	
	ESE 1	0.807			2.742
	ESE 2	0.807			3.795
	ESE 3	0.864			3.420
	ESE 4	0.882			2.302
**Entrepreneurial Intention**		0.867	0.866	0.565	
	EI 1	0.842			2.468
	EI 2	0.677			2.183
	EI 3	0.788			1.789
	EI 4	0.701			2.057
	EI 5	0.739			2.351
**Proactive Personality**		0.947	0.946	0.639	
	PP1	0.825			3.673
	PP 2	0.830			3.413
	PP 3	0.856			3.070
	PP 4	0.838			2.610
	PP 5	0.848			2.821
	PP 6	0.715			3.219
	PP 7	0.768			2.530
	PP 8	0.738			2.893
	PP 9	0.810			2.231
	PP 10	0.749			2.710
**Entrepreneurial Behavior**		0.959	0.959	0.700	
	EB 1	0.792			2.619
	EB 2	0.896			3.047
	EB 3	0.819			3.665
	EB 4	0.891			3.345
	EB 5	0.771			3.164
	EB 6	0.763			3.011
	EB 7	0.891			3.178
	EB 8	0.848			3.927
	EB 9	0.832			3.779
	EB 10	0.848			3.373

### Discriminant Validity

Discriminant validity was also measured using both criteria of the Fornell Larcker and Hetero-trait and Mono-trait ratio (HTMT). Both criteria are widely accepted and have been used by previous scholars (Henseler et al., 2016; Neneh, 2019a). According to Fornell and Larcker (1981), the criteria square root of the AVE is called discriminant validity and the values of correlations were below the output of discriminate validity. As per the criteria of the Heterotrait-Monotrait Ratio, the values must be less than 0.85 (Henseler et al., 2016). Thus, it is seen that the highest achieved HTMT value was 0.397, which was below the suggested value of 0.85. Hence, all the measurement constructs meet the criteria for discriminant validity. The values of discriminant validity were shown in Tables 2, 3.

**TABLE 2:** Fornell-Larcker Criterion.

	EA	EB	EI	EP	ESE	PP	PP^∗^EI_EB
EA	**0.797**	–	–	–	–	–	–
EB	0.281	**0.836**	–	–	–	–	–
EI	0.299	0.269	**0.752**	–	–	–	–
EP	0.330	0.247	0.290	**0.886**	–	–	–
ESE	0.399	0.378	0.345	0.372	**0.843**	–	–
PP	0.149	0.288	0.110	0.249	0.122	**0.799**	–
PP^∗^EI_EB	-0.065	0.015	-0.056	-0.201	-0.112	-0.419	0.026

*EP, entrepreneurial passion; EA, entrepreneurial alertness; ESE, entrepreneurial self-efficacy; EI, entrepreneurial intention; PP, proactive personality; EB, entrepreneurial behavior. Values in diagonals are the square root of AVE. Value below diagonals is correlations.*

**TABLE 3:** Heterotriat-Monotrait Ratio (HTMT).

	EA	EB	EI	EP	ESE	PP	PP^∗^EI_EB
EA	–	–	–	–	–	–	–
EB	0.278	–	–	–	–	–	–
EI	0.295	0.267	–	–	–	–	–
EP	0.326	0.247	0.289	–	–	–	–
ESE	0.397	0.377	0.346	0.372	–	–	–
PP	0.147	0.287	0.113	0.247	0.122	–	–
PP^∗^EI_EB	0.082	0.076	0.103	0.176	0.097	0.384	–

*EP, entrepreneurial passion; EA, entrepreneurial alertness; ESE, entrepreneurial self-efficacy; EI, entrepreneurial intention; PP, proactive personality; EB, entrepreneurial behavior.*

### Structural Model Analysis

**FIGURE 2:**
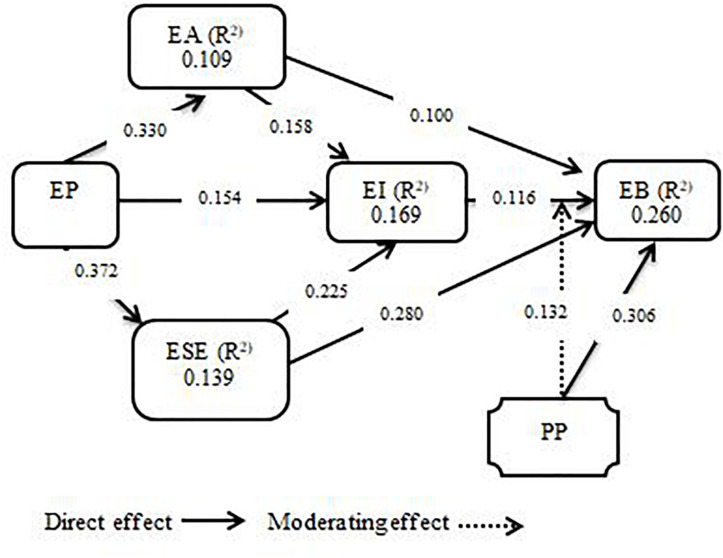
Structural Model.

The structural model was assessed using Smart-PLS software with the PLS algorithm method. The fitness of the model was evaluated with the standardized root mean square residual (SRMR) value, as suggested by Henseler et al. (2016), with the good value of SRMR at <0.08. Therefore, the value of SRMR for the proposed model was 0.048, which shows the overall fitness of the model. Furthermore, Figure 2 shows the values of *R*^2^ which explained 10.9% variance in entrepreneurial alertness, 13.9% variance in entrepreneurial self-efficacy, 16.9% variance in entrepreneurial intention, and 26.0% variance in entrepreneurial behavior. As per results from prior studies, normally, the entrepreneurial behavior-based model explained only 10–30% variance in entrepreneurial behavior (Shirokova et al., 2016; Neneh, 2019a).

The study tested the hypotheses with bootstrapping to identify the significance level between all the measurement constructs. All the results of the hypotheses were accepted, as shown in Table 4 and Figure 2. The study predicted H1 entrepreneurial passion positively influences entrepreneurial intention. The findings revealed that entrepreneurial passion has a positive and significant effect on entrepreneurial intention (β = 0.154*, *t* = 2.619, *p* < 0.01). H2 related to entrepreneurial passion and entrepreneurial alertness. The result shows that entrepreneurial passion has a positive and significance effect on entrepreneurial alertness (β = 0.330**, *t* = 5.433, *p* < 0.01). H3 explained the association of entrepreneurial passion with entrepreneurial self-efficacy. The finding shows that entrepreneurial passion has a significant and positive impact on entrepreneurial self-efficacy (β = 0.372**, *t* = 6.379, *p* < 0.01). H4 related to entrepreneurial alertness and entrepreneurial intention. The results show that entrepreneurial alertness has a positive and significance effect on entrepreneurial intention (β = 158*, *t* = 2.316, *p* < 0.01).

**TABLE 4:** Path analysis (direct effects).

Hypotheses	Relationships	Standardized Path β	t-values	*p* values	Accepted/Not Accepted
H1	EP → EI	0.154^∗^	2.619	*p* = 0.009	Accepted
H2	EP → EA	0.330^∗∗^	5.433	*p* = 0.000	Accepted
H3	EP → ESE	0.372^∗∗^	6.379	*p* = 0.000	Accepted
H4	EA → EI	0.158^∗^	2.316	*p* = 0.021	Accepted
H5	ESE → EI	0.225^∗^	3.173	*p* = 0.002	Accepted
H6	ESE → EB	0.280^∗∗^	4.427	*p* = 0.000	Accepted
H7	EA → EB	0.100^∗^	1.876	*p* = 0.049	Accepted
H8	EI → EB	0.116^∗^	1.946	*p* = 0.040	Accepted
H9	PP → EB	0.306^∗∗^	4.640	*p* = 0.000	Accepted
H10	PP^∗^EI → EB	0.132^∗^	2.886	*p* = 0.004	Accepted

*^∗∗^*p* < 0.01; *^∗^p* < 0.05. EP, entrepreneurial passion; EA, entrepreneurial alertness; ESE, entrepreneurial self-efficacy; EI, entrepreneuri.al intention; PP, proactive personality; EB, entrepreneurial behavior.*

H5 predicted the influence of entrepreneurial self-efficacy on entrepreneurial intention. Findings reveals that entrepreneurial self-efficacy has a positive and significant impact on entrepreneurial intention (β = 0.225*, *t* = 3.173, *p* < 0.01). H6 assumed the positive relationship between entrepreneurial self-efficacy and entrepreneurial behavior. The results illustrate that entrepreneurial self-efficacy has a positive and significant influence on entrepreneurial behavior (β = 0.280**, *t* = 4.427, *p* < 0.01). H7, which is associated with the impact of entrepreneurial alertness on entrepreneurial behavior, was also tested. We found that entrepreneurial alertness has a positive effect on entrepreneurial behavior (β = 0.100*, *t* = 1.876, *p* < 0.01).

H8 related to the relationship between entrepreneurial intention and entrepreneurial behavior. The findings show that entrepreneurial intention has a positive and significant effect on entrepreneurial behavior (β = 0.116*, *t* = 1.946, *p* < 0.01). H9 predicted the influence of a proactive personality on entrepreneurial behavior. The results indicate that proactive personality has a positive and significant effect on entrepreneurial behavior (β = 0.306**, *t* = 4.640, *p* < 0.01). Lastly, beyond the hypothesized relationships, it was also found that the total indirect influences of entrepreneurial self-efficacy and entrepreneurial alertness in the relationship between entrepreneurial passion and entrepreneurial intention were also positive and significant.

### Moderating Effect

**FIGURE 3:**
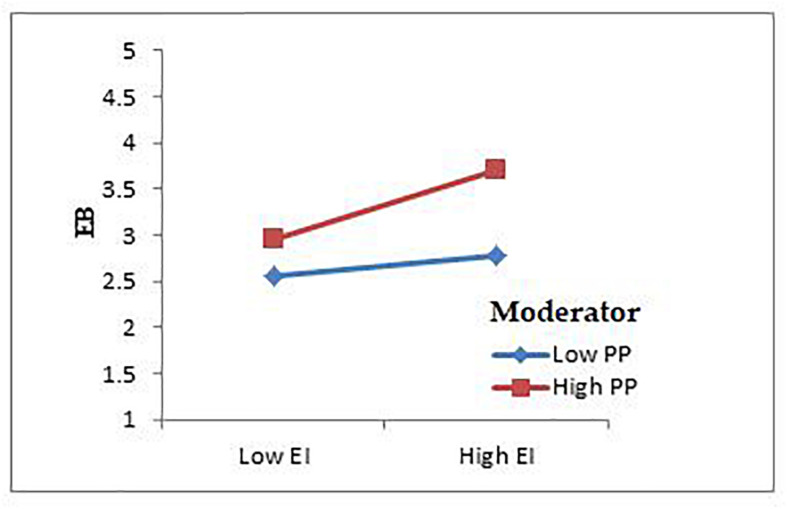
Interaction PP^∗^EI and EB.

H10 presented the moderating role of a proactive personality in the relationship between entrepreneurial intention and entrepreneurial behavior. The findings explain that a proactive personality strengthens the positive and significant relationship between entrepreneurial intention and entrepreneurial behavior (β = 0.132*, *t* = 2.886, *p* < 0.01) which is also shown in the interaction results in Figure 3.

## Discussion and Conclusion

This study aimed to investigate the role of entrepreneurial passion with entrepreneurial alertness, entrepreneurial self-efficacy, and entrepreneurial intention on entrepreneurial behavior with the moderating effect of proactive personality. The results confirmed the positive and significant contribution of entrepreneurial passion with all the indicators. The prior studies already established that entrepreneurial passion has a significant influence on entrepreneurial intention, as well as identification and exploitation of opportunities in the existing market (Cardon et al., 2009; Campos, 2017). Moreover, it was observed through previous research that entrepreneurial passion is a key influence on entrepreneurial self-efficacy and entrepreneurial intention; an individual with a high level of entrepreneurial passion is more likely to become an entrepreneur (Cardon et al., 2013; Murnieks et al., 2014).

Furthermore, existing studies discussed that entrepreneurial behavior is associated with the decision to implement ideas into reality rather than mere intentions (Kautonen et al., 2013; Shirokova et al., 2016). Lastly, previous investigations also confirmed the role of a proactive personality to strengthen the relationship between entrepreneurial intention and entrepreneurial behavior (Shinnar et al., 2018; Neneh, 2019a), because proactive personality individuals would be beneficial for shaping entrepreneurial ideas into reality and be beneficial to starting a new business.

For H1, it was demonstrated that entrepreneurial passion positively influences entrepreneurial intention, which was supported. This result is consistent with prior studies (Cardon et al., 2009; Murnieks et al., 2014; Karimi, 2020), which suggested that entrepreneurial passion energizes the entrepreneurial intention among students to become an entrepreneur. Furthermore, another justification of H1 explained that entrepreneurial passion builds a powerful desire among individuals to perform specific activities. Thus, this result is similar to previous findings (Yitshaki and Kropp, 2016; Cardon et al., 2017) that indicated the significant contribution of entrepreneurial passion in the field of entrepreneurship.

Discussing H2, it was proposed that entrepreneurial passion significantly influences entrepreneurial alertness, which was accepted. This finding is in agreement with previous research (Cardon et al., 2005; Tang et al., 2012) which suggested that entrepreneurial passion alerts individuals to the identification and exploitation of opportunities and these individuals also make essential changes to start a new business. Recent studies also support that entrepreneurial passion has a positive impact on the recognition of opportunity (Hu and Ye, 2017; Hu et al., 2018), which supports the results of this study.

Regarding H3, it was proposed that entrepreneurial passion positively affects entrepreneurial self-efficacy, which was supported. This result is consistent with earlier findings (Cardon and Kirk, 2015; Biraglia and Kadile, 2017), where it was recommended that entrepreneurial passion encourages and motivates individuals to recognize opportunities and create new businesses.

Concerning H4, it was stated that entrepreneurial alertness positively influences entrepreneurial intention, which was accepted. This finding is similar to prior studies (Obschonka et al., 2017; Neneh, 2019b; a). Entrepreneurial alertness is associated with the three dimensions: scanning and searching, information and connection, evaluation, and judgment. Researchers recognized that individuals with a high level of alertness were able to identify and recognize the opportunities and are more persuaded to start their businesses, because of their mental sharpness and recognition of appropriate opportunities in the competitive market (Van Gelderen et al., 2008; Shamsudeen et al., 2017).

Regarding H5, it was proposed that entrepreneurial self-efficacy is positively associated with entrepreneurial intention, which was supported. This result is consistent with existing studies (Naktiyok et al., 2010; Kumar and Shukla, 2019). Entrepreneurial self-efficacy refers to self-belief about a specific task to achieve the maximum results. Individuals with a high level of personal belief and self-confidence have a higher propensity to become entrepreneurs.

Concerning H6, the findings showed that entrepreneurial self-efficacy is positively influenced by entrepreneurial behavior. This hypothesis was accepted and contributes to the literature of entrepreneurship, because few studies have examined the direct role of entrepreneurial self-efficacy on entrepreneurial behavior. Individuals with a high level of self-efficacy are more inclined to accomplish a certain level of task and are also more prone to start a new business. Therefore, our results are consistent with prior research (Tsai et al., 2016; Darmanto and Yuliari, 2018) which indicated that entrepreneurial self-efficacy had a positive impact on entrepreneurial intention, which leads to entrepreneurial behavior.

Regarding H7, the results demonstrated that entrepreneurial alertness positively influenced entrepreneurial behavior. This hypothesis was accepted. Moreover, studies investigated the role of entrepreneurial alertness on entrepreneurial intentions and entrepreneurial behavior were neglected. A recent study examined the entrepreneurial behavior model using entrepreneurial alertness as a predictor and entrepreneurial intention as an outcome variable, but did not test the relationship between entrepreneurial alertness on entrepreneurial behavior (Obschonka et al., 2017; Neneh, 2019a). Therefore, to extend the previous model, we have tested the relationship of entrepreneurial alertness on entrepreneurial behavior and found significant results.

Concerning H8, it was predicted that entrepreneurial intention positively related to making entrepreneurial behavior, and this was accepted. This finding is similar to previous studies (Shirokova et al., 2016; Shinnar et al., 2018; Bogatyreva et al., 2019; Yi, 2020). Entrepreneurial behavior refers to taking actions, not mere intentions; it is necessary to translate these intentions into reality or action to create entrepreneurial behavior.

Relating to H9, it was predicted that proactive personality significantly influences entrepreneurial behavior, which is supported. This result is consistent with earlier studies (Zampetakis, 2008; Delle and Amadu, 2016; Hu et al., 2018). Proactive personality is associated with the active attempt made by the individual to affect his or her dynamic environment. Therefore, individuals with proactive personalities are inclined to take the best initiative to the environment stimulus; it would also be beneficial to students that have already developed an intention to become an entrepreneur.

Regarding H10, it was proposed that proactive personality moderated the relationship between entrepreneurial intention and entrepreneurial behavior, which was accepted and strengthened the connection. This finding is similar to the recent studies of Neneh, 2019b; a which indicated that a proactive personality broadens entrepreneurial intention and translates into entrepreneurial behavior effectively and efficiently.

The present study suggests some theoretical and practical implications for academicians, scholars, and policymakers. This study highlights the significant influence of entrepreneurial passion, entrepreneurial alertness, and entrepreneurial self-efficacy on entrepreneurial intention with the moderation effect of proactive personality in the formation of entrepreneurial behavior. This study contributes to the role of SCT theory among these indicators to better predict the entrepreneurial intention of individuals (Bandura, 1982; Biraglia and Kadile, 2017). Furthermore, previous findings on entrepreneurial passion and entrepreneurial alertness on entrepreneurial intention have been diversified; precisely, entrepreneurial passion and entrepreneurial alertness with entrepreneurial intention become non-significant due to the conceptual measurement model (Demmert and Klein, 2003; Kitzmann and Schiereck, 2005).

Therefore, this study provides a positive contribution to strengthen the relationship between entrepreneurial passion, entrepreneurial alertness, and entrepreneurial self-efficacy on entrepreneurial intention, as well as the role of proactive personality, while translating the intentions into actions. Moreover, existing studies claimed that entrepreneurial intentions are not convertible into entrepreneurial behavior due to different individual circumstances and environmental conditions (Shirokova et al., 2016; Shinnar et al., 2018). Likewise, this study contributes to the moderating role of proactive personality, which strengthens the association between entrepreneurial intention and entrepreneurial behavior.

The study has some practical implications for governmental education sectors, NGO’s, and institutions that are currently working in the development of student entrepreneurial skills and abilities. Firstly, previous studies focused on entrepreneurial intentions and were less focused on entrepreneurial behavior. Therefore, this study provides new ways of motivating individuals to move forward from entrepreneurial intention to energetic steps to become entrepreneurs. Secondly, entrepreneurial passion develops entrepreneurial alertness among individuals. Therefore, individuals with a high level of entrepreneurial passion are more likely to be motivated by the recognition and exploitation of opportunity as well as act on their entrepreneurial intentions. Thus, it is necessary to find the entrepreneurial passion among students and accomplish their talents through different training and seminars to translate it in the creation of opportunity recognition and entrepreneurial behavior. Thirdly, previous literature claimed that a high level of entrepreneurial intentions among individuals was not successfully translated into entrepreneurial actions due to different environmental situations, hence the proactive personality motivating them to translate their entrepreneurial intention into action. Fourthly, entrepreneurial intentions are essential for developing the entrepreneurial process and shaping this into entrepreneurial behavior.

Moreover, this study indicated that entrepreneurial passion and entrepreneurial self-efficacy positively influence entrepreneurial intention. As such, entrepreneurial intentions can be bettered by an individual’s self-belief to create a new business effectively and efficiently. Currently, Chinese universities are focusing on entrepreneurship education as a means to enhance entrepreneurial intentions and successful entrepreneurial behavior. Therefore, it is essential to confirm that such programs take into attention with special regards to entrepreneurial passion, entrepreneurial alertness, entrepreneurial self-efficacy, and proactive personalities. Lastly, universities encourage students to start new businesses and contribute to the economic development of the country and society. Entrepreneurial passion and personality development must be a compulsory course topic in the curriculum of entrepreneurship and all the educational streams. Consequently, it is proposed that academic institutions must encourage students to participate in entrepreneurial seminars and live projects, and formulate an innovative business plan during the course of their program. Finally, management must be focused on the features of entrepreneurship among students and make an industrial liaison to facilitate their students with innovative technology adoption regarding the enhancement of entrepreneurship study.

### Limitations and Future Directions

The present study has some limitations and future directions for upcoming researchers. Firstly, this study has taken indicators such as entrepreneurial passion, entrepreneurial alertness, entrepreneurial self-efficacy, and proactive personality to investigate the entrepreneurial intention and entrepreneurial behavior of students. This study mainly focused on management, finance and economics, and public administration departments of undergraduate, masters, and Ph.D. students from Zhenjiang city, Jiangsu province, China, with a small sample size. The nature of our study was cross-sectional, and data were collected through a self-report questionnaire, creating some potential inherent problems; therefore, an assessment of common method bias was made.

Secondly, this study used Smart-PLS software to investigate the variance base relationship between all the measurement constructs. Moreover, the study strongly suggests to upcoming researchers to conduct a longitudinal design study on different samples with these indicators to contribute more to the literature of entrepreneurship. This study also recommends future researchers incorporate the investigation of other factors of entrepreneurship, e.g., alertness, risk-taking propensity, family, and social support, with entrepreneurial intention or entrepreneurial self-efficacy. Future researchers may also employ these constructs on different samples, e.g., SME’s sector entrepreneurs to enhance their firm performance with entrepreneurial orientation.

## Data Availability Statement

The datasets generated for this study are available on request to the corresponding author.

## Ethics Statement

The review board of Jiangsu University exempted the research from ethical approval as it was a survey-based study. The patients/participants provided their written informed consent to participate in this study.

## Author Contributions

MM and CL conceived the study idea, edited the data, performed the analysis and interpretation, drafted the skeleton of the manuscript, and critically reviewed the manuscript. FS, MK, SA, and CD contributed to constructing the model, interpretation of model results, and intensively editing the language of the manuscript. All authors read and approved the final manuscript and participated in the critical appraisal as well as revision of the manuscript.

## Conflict of Interest

The authors declare that the research was conducted in the absence of any commercial or financial relationships that could be construed as a potential conflict of interest.

## Appendix

**TABLE A1:** Questionnaire.

**Entrepreneurial Passion**	
EP 1	It is exciting to figure out new ways to solve unmet market needs that can be commercialized
EP 2	Searching for new ideas for products/services to offer is enjoyable to me
EP 3	I am motivated to figure out how to make existing products/services better
EP 4	Scanning the environment for new opportunities really excites me
EP 5	Inventing new solutions to problems is an important part of who I am
**Entrepreneurial Alertness**	
**Scanning and Search**	
SS 1	I have frequent interactions with others to acquire new information
SS 2	I always keep an eye out for new business ideas when looking for information
SS 3	I read news, magazines, or trade publications regularly to acquire new information
SS 4	I browse the internet every day
SS 5	I am an avid information seeker
SS 6	I am always actively looking for new information
**Association and Connection**	
AC 1	I see links between seemingly unrelated pieces of information
AC 2	I am good at “connecting dots”
AC 3	I often see connections between previously unconnected domains of information
**Evaluation and Judgment**	
EJ 1	I have a gut feeling for potential opportunities
EJ 2	I can distinguish between profitable opportunities and not-so- profitable opportunities
EJ 3	I have a knack for telling high-value opportunities apart from low-value opportunities
EJ 4	When facing multiple opportunities, I am able to select the good ones.
**Entrepreneurial Self-Efficacy**	
ESE 1	I am convinced that I can successfully discover new business opportunities
ESE 2	I am convinced that I can successfully create new products
ESE 3	I am convinced that I can think creatively
ESE 4	I am convinced that I can successfully commercialize ideas
**Entrepreneurial Intention**	
EI 1	I am ready to do anything to be an entrepreneur
EI 2	My professional goal is to become an entrepreneur
EI 3	I will make every effort to start and run my own firm
EI 4	I am determined to create a firm in the future
EI 5	I have the firm intention to start a firm someday
**Proactive Personality**	
PP 1	I am constantly on the lookout for new ways to improve my life
PP 2	Wherever I have been I am a powerful force of constructive change
PP 3	Nothing is more exciting than seeing my ideas turn in to reality
PP 4	If I see something I don’t like, I fix it
PP 5	No matter what the odds, If I believe in something, I will make it happen
PP 6	I love being a champion for my ideas, even against other’s opposition
PP 7	I excel at identifying opportunities
PP 8	I am always looking for better ways to do things
PP 9	If I believe in an idea, no obstacle will prevent me from making it happen
PP 10	I can spot a good opportunity long before others can
**Entrepreneurial Behavior**	
EB1	I have discussed product or business idea with potential customers
EB2	I have collected information about markets or competitors
EB3	I have written a business plan
EB4	I have started product/service development
EB5	I have started marketing or promotion efforts
EB6	I have purchased material, equipment, or machinery for the business
EB7	I attempted to obtain external funding
EB8	I have applied for a patent, copyright, or trademark
EB9	I will register the company
EB10	I will sell products or services
